# A micro-Raman study of exfoliated few-layered *n*-type Bi_2_ Te_2.7_Se_0.3_

**DOI:** 10.1038/s41598-017-16479-y

**Published:** 2017-11-28

**Authors:** Fengjiao Liu, Longyu Hu, Mehmet Karakaya, Pooja Puneet, Rahul Rao, Ramakrishna Podila, Sriparna Bhattacharya, Apparao M. Rao

**Affiliations:** 1 Clemson Nanomaterials Institute, Department of Physics & Astronomy, Clemson University, Clemson, 29634 SC USA; 2 Department of Chemistry, Clemson University, Clemson, SC 29634 USA; 3 Materials and Manufacturing Directorate, Air Force Research Laboratory, WPAFB, Dayton, OH 45433 USA; 4 UES Inc., Dayton, OH 45432 USA; 5 Laboratory of Nano-biophysics, Clemson University, Clemson, SC 29634 USA

## Abstract

Previously we showed that the thermoelectric (TE) performance of bulk *n*-type Bi_2_Te_2.7_Se_0.3_ can be enhanced by subjecting it to a combined process of chemical or mechanical exfoliation (C/ME) followed by a rapid densification and restacking of the exfoliated layers via the spark-plasma-sintering technique (SPS). Here, we present a systematic micro-Raman study of two-dimensional flakes of *n*-type Bi_2_Te_2.7_Se_0.3_ produced by the C/ME process, as a function of the flake thickness. We found Raman evidence for flakes with: (i) integer number of quintuples which exhibited a strong electron-phonon coupling, and (ii) non-integer number of quintuples, or sub-quintuples which exhibited the forbidden IR active mode due to symmetry lowering. Detailed atomic force microscopy was used to confirm the number of quintuples in all flakes examined in this study. The restacking and densification of these flakes by SPS promoted the formation of charged grain boundaries, which led to the enhanced TE properties via the energy filtering process.

## Introduction

Bulk pristine (undoped) and doped Bi_2_Te_3_ are some of the most efficient room temperature thermoelectric (TE) materials for sustainable power generation and refrigeration applications^1–3^. The efficiency of a TE material is determined by its dimensionless figure of merit, *ZT* = *α* ^2^ *σT*/*κ*, where *α* is Seebeck coefficient or thermopower, *σ* is electrical conductivity, and *κ*( = *κ*_E_ + *κ*_L_) is the total thermal conductivity, which is comprised of electronic and lattice contributions, respectively. The main challenge for improving *ZT* of any TE material is the inherent coupling between *α*, *σ*, and *κ* that prevents increased *α* and *σ* with a concomitant decrease in *κ*. Bi_2_Te_3_ is also of tremendous interest as a topological insulator (TI), which in turn is promising for TE energy conversion^4–6^. In contrast to ordinary materials, backscattering of electrons due to collisions with impurities and defects in the crystal lattice is completely suppressed on the surfaces of TIs, giving rise to improved charge transfer and mobility, and thus enhanced *σ*. The existence of surface states in TIs arise from the intrinsic spin-orbit coupling that is enhanced with increasing atomic masses, which can also increase the mass fluctuation scattering effect to reduce *κ*_*L*_. It is thus observed that most topological insulators such as Bi_2_Te_3_, Bi_2_Se_3_, exhibit excellent TE properties.

In the 1990s, Hicks and Dresselhaus predicted that an increase in the density of states could lead to enhanced α^7^. In addition to improving electronic transport in low dimensional materials, nanostructuring via ball-milling and melt-spinning have been effective in reducing *κ*_*L*_ through increased phonon scattering effects, resulting in an enhanced *ZT* in TE nanomaterials^8,9^. In recent years, chemical/mechanical exfoliation (C/ME) of layered materials has enabled the fabrication of two-dimensional (2D) nanosheets that exhibit superior TE properties compared to their bulk counterparts^10–14^.

Bulk Bi_2_Te_3_ exhibits a layered crystal structure and its conventional unit cell is comprised of three quintuples. Each quintuple contains five atomic layers with a sequence of Te^1^-Bi-Te^2^-Bi-Te^1^ called quintuple layers (QL), and the weak van der Waals bond between Te^1^-Te^1^ couples the quintuples together^15^. Therefore during the C/ME process, the Te^1^-Te^1^ bond between the QLs can be broken, giving rise to unique optical and electronic transport, compared to the bulk^16,17^. Previously we employed the combined technique of C/ME followed by spark-plasma-sintering (SPS), which led to preferential scattering of electrons at charged grain boundaries, and significantly improved the TE compatibility factor and stabilized the *ZT* peak at higher temperatures (350–500 K) in *n*-type Bi_2_Te_2.7_Se_0.3_^10^. The C/ME-SPS process resulted in two important contributions: (i) an increase in the electrical conductivity due to an increase in carrier concentration despite the presence of numerous grain boundaries, and (ii) the mitigation of the bipolar effect via band occupancy optimization which led to an upshift and stabilization of the *ZT* peak over a broad temperature range of ~150 K^10^. Both (i) and (ii) implied the creation of charged grain boundaries in Bi_2_Te_2.7_Se_0.3_ due to the C/ME-SPS process.

To understand the structural changes in the Bi_2_Te_2.7_Se_0.3_ brought about by the C/ME process, we performed a detailed micro-Raman study of C/ME *n*-type Bi_2_Te_2.7_Se_0.3_ with varying layer thicknesses down to ~2 nm (~2 quintuples). To the best of our knowledge, previous Raman studies of bulk and few-layered Bi_2_Te_3_ delineated the Raman-active modes in pristine Bi_2_Te_3_, and the effect of Se doping in the chemically exfoliated layers was still largely ignored. Our transport measurements were performed on the exf*nh*-SPS samples (exf*nh*, where *n* represents the exfoliation time in hours) and showed a decrease in electrical resistivity concomitant with an increase in carrier concentration, thereby resulting in a lower Seebeck coefficient. Moreover, we found a decrease in thermal conductivity with decreasing layer thickness. Micro-Raman measurements on the C/ME samples revealed the co-existence of whole quintuples with strong surface states (electron-phonon coupling) and sub-quintuples that exhibited forbidden (IR-active) modes in the Raman spectra. The combination of these two “phases” results in the formation of charged grain boundaries upon SPS-processing and thus enhanced TE performance.

## Results and Discussion

Bulk Bi_2_Te_3_ exhibits a trigonal crystal structure belonging to the space group R$\overline{3}$ m^18^, but is more commonly represented by a hexagonal crystal structure (see Supplementary Fig. S1). The highly anisotropic hexagonal unit cell consists of three quintuples each consisting of five atoms, stacked in the order Te^1^-Bi-Te^2^-Bi-Te^1^ along the *c*-axis, with lattice constants *a* = 4.38 Å and *c* = 30.36 Å^1^. Each quintuple measures approximately 1 nm across the five atoms. The quintuples are held together by weak van der Waals forces (Te^1^-Te^1^ bond) corresponding to the largest spacing *d* ~ 0.37 nm^16^ that make them easily cleavable. In general, for Bi_2_Te_3−*x*_Se_*x*_ the Se atoms preferentially replace Te at Te^2^ sites first and then randomly replace Te at the Te^1^ sites^14,19^. With Se-doping at the Te^2^ sites, a breakdown of the Bi_2_Te_2.7_Se_0.3_ quintuples into bi-layer or tri-layer sub-quintuples (e.g., Bi-Te^1^, Te^1^-Bi-Te^2^, Te^1^-Bi-Se^2^) during the C/ME process is feasible because the Bi-Te^1^ bond strength is the strongest bond in the quintuple^20^. In addition, the Te^2^ atom is known to lie at the inversion center of the D_3*d*_^5^ symmetry^21^, and hence the Se doping can change the crystalline structure as well as the lattice dynamics in a unique manner.

**Figure 1:**
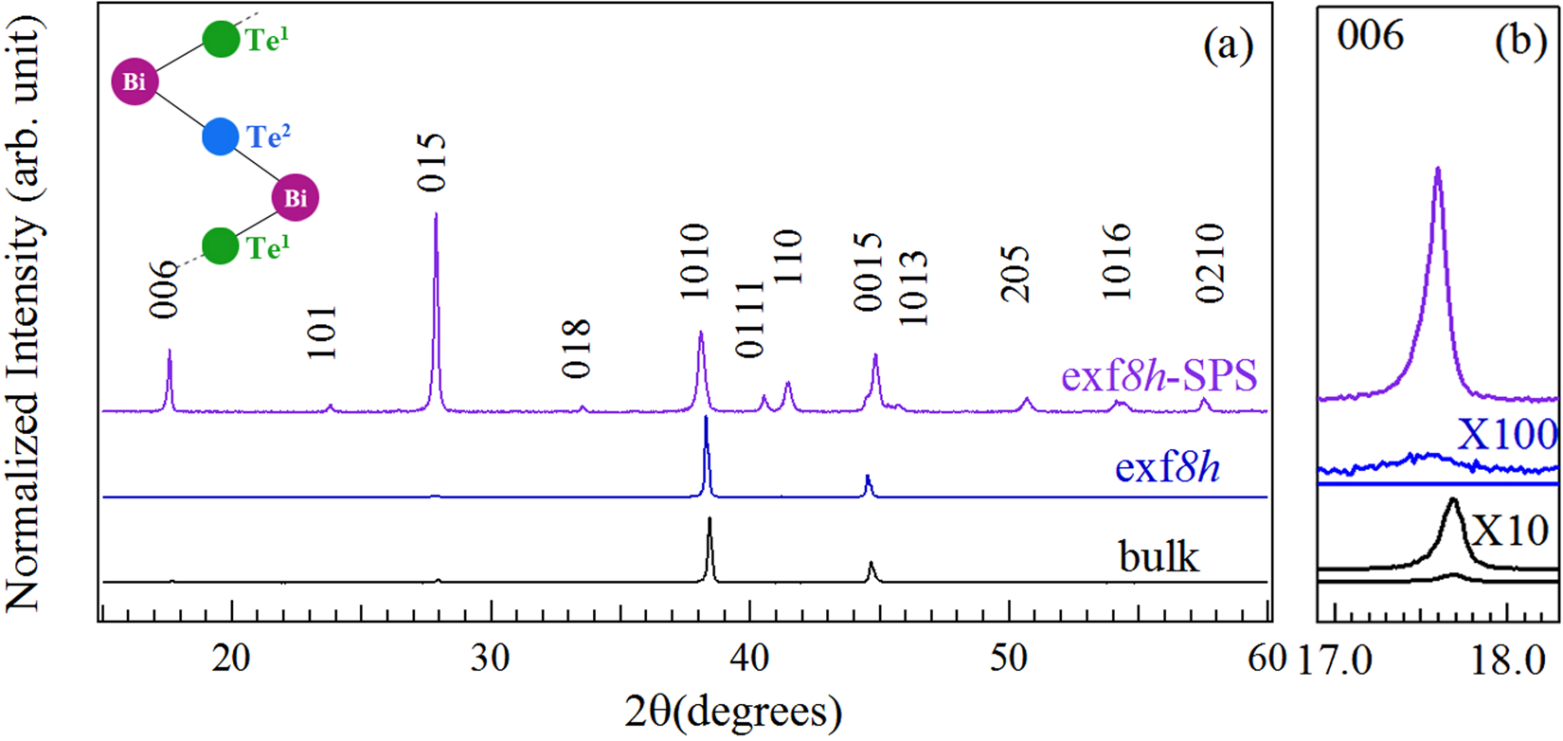
X-ray diffraction patterns. (**a**) Bulk *n*-type Bi_2_Te_2.7_Se_0.3_ sample compared to the 8-hr exfoliated flake before and after SPS with a Bi_2_Te_3_ quintuple as inset; (**b**) (006) peak suppressed in the exf*8h* (X100), but more pronounced in the exf*8h*-SPS.

**Figure 2:**
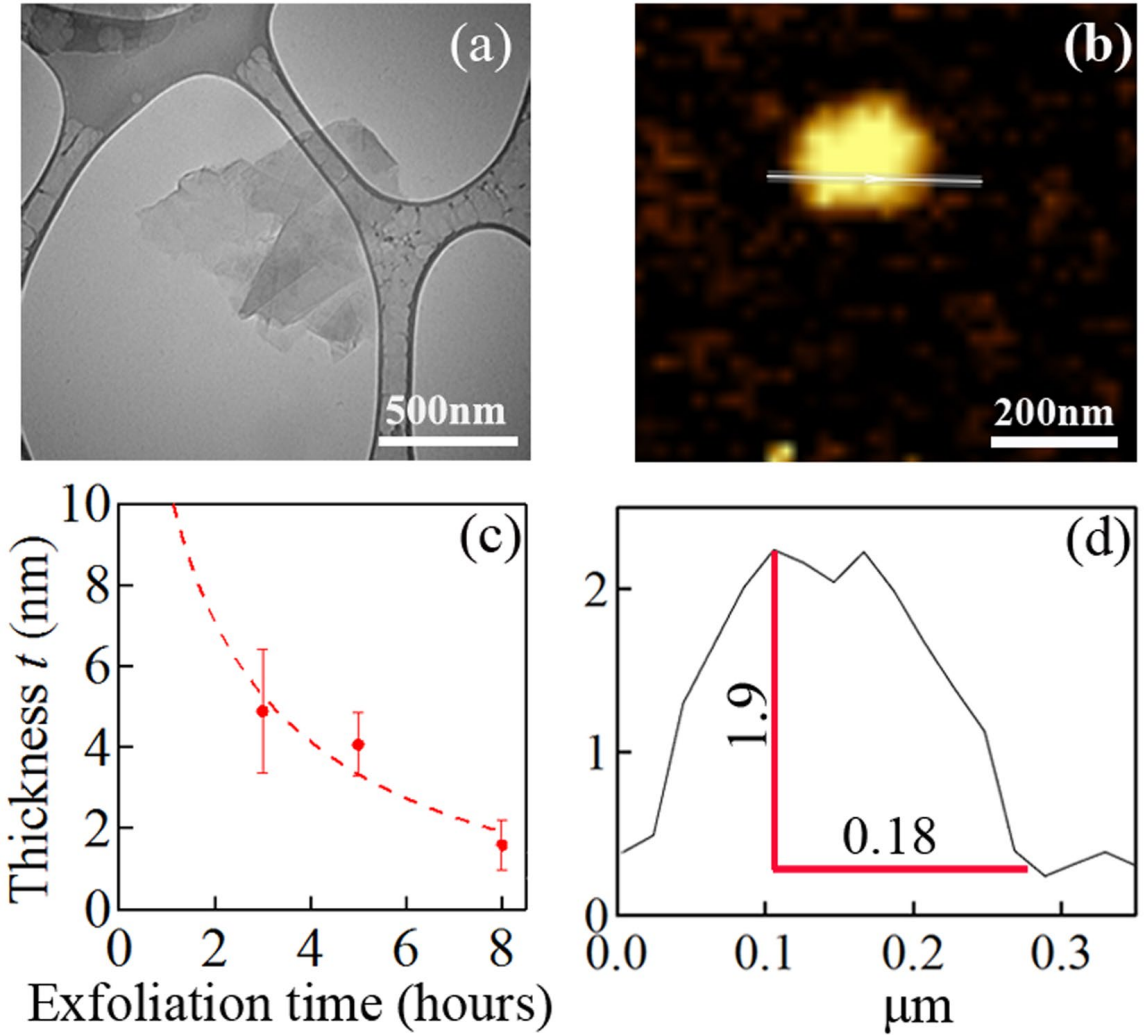
Representative TEM (**a**) and AFM (**b**) images of the exf*8h* sample. (**c**) The average thickness of the exfoliated *n*-type Bi_2_Te_2.7_Se_0.3_ flakes as a function of exfoliation times (0 h, 3 h, 5 h, and 8 h). The full width of half maximum of thickness distribution (Gaussian distribution) were used as error bars. *t* tends to be infinite at 0 h and the dash line is a guide to the eye. (**d**) The lateral size and height of a representative flake shown in (**b**).

Figure 1a shows the powder HR-XRD patterns of the bulk compared to that of the exf*8h* and exf*8h*-SPS samples. Both XRD patterns are consistent with the expected pattern for Bi_2_Te_2.7_Se_0.3_ (JCPDS card no. 00-050-0954). The (006) peak (Fig. 1b) was severely broadened in the data of the exf*8h* sample (inferred by magnifying it a 100 fold) due to nano-structuring and bond cleavages at various locations along the *c*-direction, while it became more pronounced after SPS treatment, suggesting an improvement in the coherence length. The representative TEM and AFM images (Fig. 2a and b) of the exf*8h* samples show lateral sizes ranging from ~0.3 *μ*m to 0.8 *μ*m and a height of ~2 nm (Fig. 2d). The roughness (R_*q*_)^22^ of this representative AFM image is about 0.1 nm. The average thickness of the *n*-type Bi_2_Te_2.7_Se_0.3_ layers as a function of the exfoliation time is shown in Fig. 2c. With an increase in the exfoliation time, the layer thickness *t* of the Bi_2_Te_2.7_Se_0.3_ decreased significantly between the exfoliation times of 0 to 3 hr, down to ~1.6 nm after 8 hr exfoliation. Further exfoliation time did not significantly reduce the thickness of the flakes. The presence of sub-quintuples was evidenced from the non-integer values of *t* from AFM linescans of the flakes, since 1 nm corresponds to one quintuple as discussed above.

**Figure 3:**
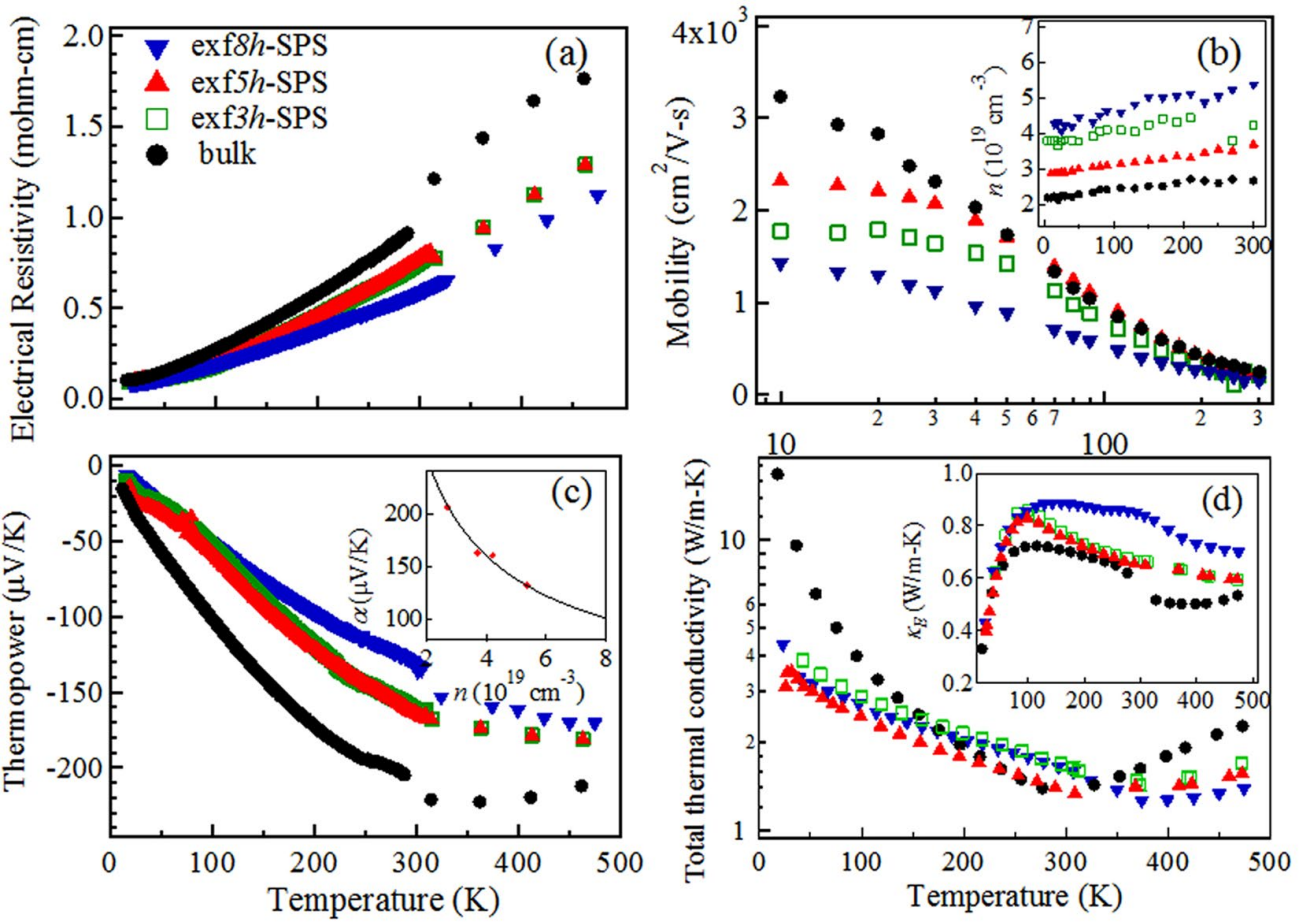
Temperature dependence of transport properties. (**a**) The electrical resistivity (*ρ*); (**b**) mobility (*μ*) with carrier concentration (*n*) as inset; (**c**) temperature dependence of the Seebeck coefficient (*α*) with its dependence on carrier concentration shown in the inset; (**d**) total thermal conductivity (*κ*) with electronic thermal conductivity (*κ*_*E*_) as inset of the exf*3h*-SPS, exf*5h*-SPS, exf*8h*-SPS samples as a function of temperature compared to the bulk sample.

Figure 3 shows the thermoelectric transport properties of the exf*nh*-SPS samples compared to the bulk sample. The electrical resistivity (*ρ* = 1/*σ*) increased linearly with increasing temperature (Fig. 3a), indicating a “metal-like” or degenerate semiconducting behavior. A slight change of slope was observed in the bulk sample at ~360 K, consistent with the upturn in *α* (Fig. 3c) due to the bipolar effect. With increasing exfoliation time, the *ρ* values in the exf*nh*-SPS samples were consistently lower than the corresponding value in the bulk due to increase in the carrier concentration *n* (Fig. 3b inset). The carrier mobility *μ* = 1/*neρ*, where *e* is the electron charge, (Fig. 3b) also exhibited a reduction with increased exfoliation times at temperatures below 100 K where the grain boundary and charged defect scattering dominates. Puneet *et al*. attributed the increase in *n* to selective filtering of charge carriers by positively charged grain boundaries resulting from *Te*_*Bi*_ anti-site and bismuth vacancies (*V*_*Bi*_ defects)^10,16^.

The Seebeck coefficient or thermopower (*α*) of the bulk Bi_2_Te_2.7_Se_0.3_ sample (Fig. 3c) exhibited a high negative value (~200 *μ*V/K) at 300 K, while *α* in the exf*nh*-SPS samples reduced to 130~170 *μ*V/K (65~85% of the bulk value), which is consistent with the reduction of *ρ* due to increase in *n*, as *α* varies as *n* ^−2/3^ shown in equation (1). Using a simple parabolic band approximation, the effective masses (m*) of the charge carriers in exf*nh*-SPS samples were calculated from the measured *α* and *n* values at room temperature using the relation^23,24^.

1 $$\alpha =(\frac{8{\pi }^{2}{k}_{B}^{2}}{3e{h}^{2}}){m}^{\ast }T{(\frac{\pi }{3n})}^{(2/3)}$$

where *k*_*B*_ is the Boltzmann constant, *e* the electronic charge, and *h* the Planck’s constant. The effective masses (m*) for the bulk and exf*nh* samples, estimated from *α* vs. *n* at 300 K (Pisarenko plot, Fig. 3c inset) were found to be ~0.95 *m*_*e*_, where *m*_*e*_ is the mass of the electron. In addition, the band gaps (*E*_*g*_) of the exfoliated samples estimated using the Goldsmid Sharp relation^25^:

2 $${E}_{g}=2e{\alpha }_{{\max }}{T}_{{\max }}$$

remained constant ~0.16 eV (see Supplementary Table S1) which are consistent with the reported values^1^. This indicates that the systematic reductions in *α* with increasing exfoliation times could be solely attributed to increasing *n* (inset, Fig. 3b) and not from changes in band gap or band curvature. With increasing temperature, |*α*| of the bulk sample increased till *α*_*max*_ was reached, above which the bipolar (two carrier) effect was observed. Specifically, |*α*| decreased as indicated by the upturn at T = 362 K in Fig. 3c, which is typical of a narrow band gap semiconductor. As concluded by Puneet *et al*. the low energy minority carriers in the exf*8h* samples were selectively filtered due to the presence of the positively charged defects on the grain boundary (interfacial charged defects) which upshifted the *α*_*max*_ to above 500 K^10^. With the reduction of the bipolar term, this upshift in *α*_*max*_ led to the broadening of *ZT* in the exf*nh*-SPS samples as shown in Supplementary Fig. S2.

The presence of grain boundary potential barrier scattering in the *n*-type Bi_2_Te_2.7_Se_0.3_ samples is evidenced from the temperature dependence of *σ*, where $\sigma \sim {T}^{-1/2}\exp (\frac{-{E}_{B}}{kT})$ ^26^ and the associated grain boundary potential barrier height (E_*B*_) was calculated from the linear plot of *ln*(*σT* ^1/2^) *vs. 1/kT* as shown in Supplementary Fig. S3. As expected, the exf*nh*-SPS samples exhibited higher E_*B*_ ~ 23–27 meV arising from the increased number of grain boundaries (grain size ~20 *μ* m) compared to the E_*B*_ ~ 19 meV of the ingot that exhibited relatively larger grains. For comparison, an E_*B*_ ~ 60 meV was reported in PbTe nanocomposites, wherein grain boundary potential barrier scattering is known to be a dominant scattering mechanism^27^. Furthermore, evidence for the presence of charged grain boundaries in *n*-type Bi_2_Te_2.7_Se_0.3_ samples was also found using Kelvin probe Force Microscopy (KPFM; Supplementary Fig. S4) as detailed in the SI section.

The bulk sample exhibited a high magnitude of total thermal conductivity (*κ*) at ~17 K as shown in Fig. 3d, which decreased subsequently in the exf*nh*-SPS samples due to exfoliation-induced disorder. A well-defined peak is expected at lower temperatures in the bulk sample (indicating good crystal quality), before *κ* decreased with increasing temperature due to anharmonic phonon-phonon or Umklapp scattering effects. At ~360 K, *κ* increased gradually with increasing temperature, due to contribution from the bipolar effect. The inset showed the systematic increase in *κ*_*E*_ (=*L*_0_ *σT*) (where *L*_0_ is the Lorenz number, *L*_0_ = 1.66 × 10^−8^ V^2^/K^2^ for the nanostructured samples^28^) in the exf*nh*-SPS samples compared to the bulk, indicating that *κ*_*T*_ − *κ*_*E*_ was also reduced due to increased phonon scattering at the grain boundaries. The bipolar contribution however was absent and possibly shifted to higher temperatures in the exfoliated samples. Taken together, the plots in Fig. 3 show that the C/ME-SPS treatment of *n*-type resulted in: i) an increase (decrease) in carrier concentration (resistivity), ii) mitigation of the bipolar effect in thermopower, and iii) a simultaneous reduction in the thermal conductivity, that led to the broadening of the *ZT* peak over a wider range of temperature ~100 K (see Supplementary Fig. S2).

To gain more understanding into the structural properties underpinning the unique TE performance of the exfoliated samples, we used micro-Raman spectroscopy, which is an ideal technique for studying the changes in the vibrational (and hence TE) properties caused by the C/ME process in *n*-type Bi_2_Te_2.7_Se_0.3_. Micro-Raman spectra were collected from several different spots on the bulk, exf*3h*, exf*5h* and exf*8h* Bi_2_Te_2.7_Se_0.3_ samples. Upon densifying these samples using SPS, the SPS-compacted samples exhibited a high background making them unsuitable for Raman studies. Nevertheless, significant structural changes in the exfoliated layers due to the C/ME process were evident in our micro-Raman spectra, as discussed below.

**Figure 4:**
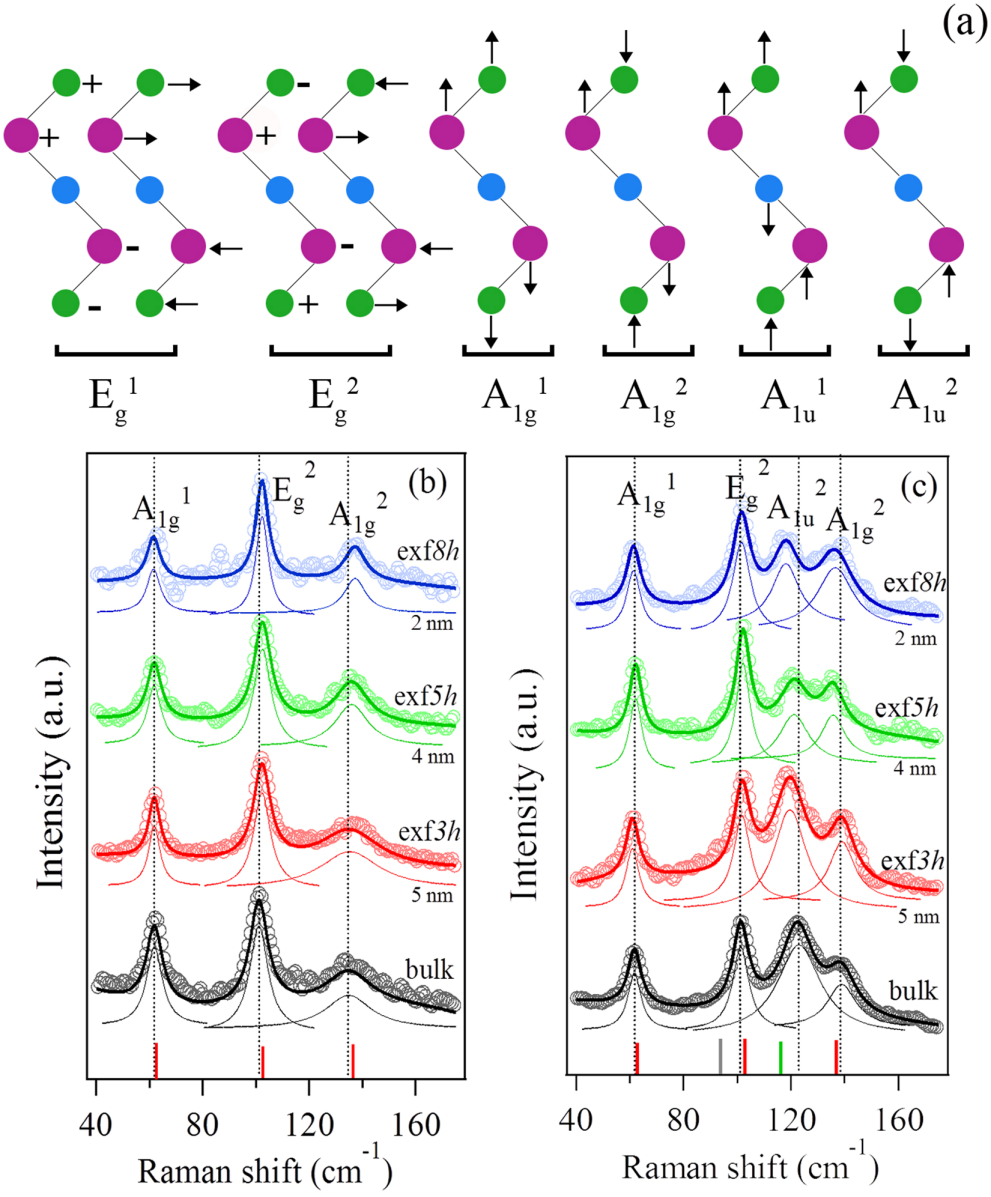
Vibration modes and Raman measurement. (**a**) Schematic diagram of the four Raman-active modes and two IR-active modes of the Bi_2_Te_3_ quintuple. The purple, blue and green colors represent the Bi, Te^2^ and Te^1^ atoms respectively. The vertical arrows represent out of plane vibration. The horizontal arrows represent in plane vibration that parallel to the page. The −, + signs represent in plane vibrations that are perpendicular to the page. (**b**,**c**) Micro-Raman spectra of *n*-type Bi_2_Te_2.7_Se_0.3_ without and with the presence of the A_1*u*_^2^ mode as function of different exfoliation times compared to the bulk sample. The open symbols represent the raw data and the solid lines through the raw data are the fits. The individual Lorentzian fits are shown below each spectrum. The red bars indicate the position of the optical phonon peaks of bulk *n*-type Bi_2_Te_2.7_Se_0.3_. The gray (green) bar in (**c**) indicate the peak position of the IR-active A_1*u*_^1^ (A_1*u*_^2^) mode in bulk Bi_2_Te_3_.

Bulk *n*-type Bi_2_Te_2.7_Se_0.3_ is known to exhibit four signature Raman-active optical phonons as shown in Fig. 4. Of these four modes, the lowest frequency E_*g*_^1^ mode was difficult to resolve owing to the increasing spectral background below 40 cm^−1^. The other three modes^21^ are centered at around 62.4 cm^−1^ (A_1*g*_^1^), 102.6 cm^−1^ (E_*g*_^2^) and 136.5 cm^−1^ (A_1*g*_^2^) as represented by the red vertical bars in the spectra in Fig. 4b and c. Two types of Raman spectra were observed at various spots from all samples irrespective of the exfoliation time: those that exhibited the three modes mentioned above (Fig. 4b), and those that exhibited an additional peak at ~122 cm^−1^ (Fig. 4c). The additional peak is an IR-active mode (A_1*u*_^2^) that has been reported previously^16,29–32^, in nanoscale pristine Bi_2_Te_3_ (mode at around 116 cm^−1^ indicated by the green vertical bar in Fig. 4c). We attribute the presence of the A_1*u*_^2^ mode to symmetry breaking, possibly arising from the disorder induced by exfoliation and/or Se-dopant at the Te sites (Te^1^ and Te^2^), where Te^2^ is the inversion center of the crystal symmetry (see Supplementary Fig. S1)^33^. As Se is lighter than Te, A_1*u*_^2^ mode was found blueshifted to ~122 cm^−1^ compared to the corresponding peak frequency in bulk Bi_2_Te_3_ (Fig. 4c), confirming the presence of Se. Several dozen spots were scanned, and A_1*u*_^2^ mode was observed in the Raman spectra at roughly half of these spots, as can be seen in the 2D Raman intensity maps in supplementary Fig. S5. The random occurrence of the A_1*u*_^2^ mode suggests variations in the structure of the exfoliated sheets, possibly from cleaving of the layers into sub-quintuples, as discussed further below. The other low wave number IR-active mode (A_1*u*_^1^) at ~94 cm^−1^ ^34^ (mode indicated by the gray vertical bar in Fig. 4c) in Bi_2_Te_3_ could not be discerned as this peak’s frequency is close to that of the E_*g*_^2^ mode. Moreover, the A_1*u*_^1^ mode frequency may have blueshifted and as a result could be masked by the E_*g*_^2^ mode.

**Figure 5:**
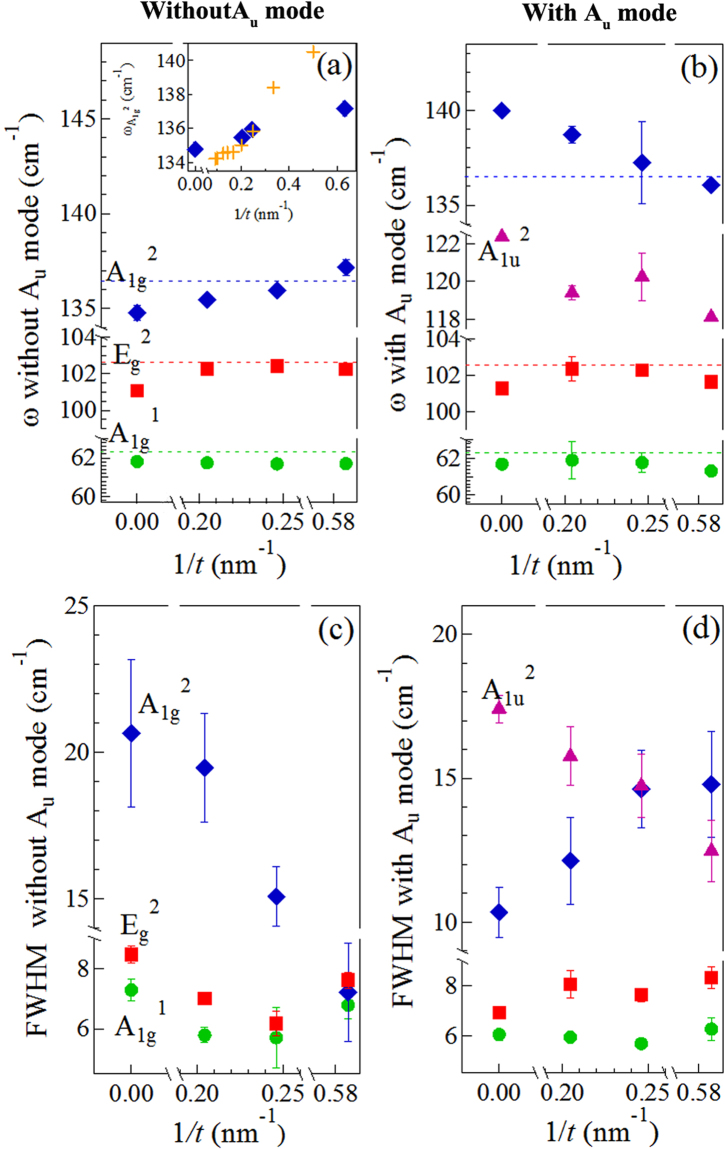
Frequency and FWHM dependence of 1/*t*. (**a**) Dependence of the A_1*g*_^1^, E_*g*_^2^, A_1*g*_^2^ mode frequencies as a function of 1/*t* when the A_*u*_ mode is absent. Inset compares the A_1*g*_^2^ mode frequency (+) from Zhao *et al*.^35^ with current work. (**b**) Dependence of the A_1*g*_^1^, E_*g*_^2^, A_1*g*_^2^ and A_1*u*_^2^ mode frequencies as a function of 1/*t*. The dashed horizontal lines in (**a**) and (**b**) represent the peak frequencies of Raman modes in bulk *n*-type Bi_2_Te_2.7_Se_0.3_. Change in linewidths (FWHM) of the A_1*g*_^1^, E_*g*_^2^, A_1*g*_^2^ modes as a function of 1/*t* (**c**) when the A_1*u*_^2^ mode is absent, (**d**) when the A_1*u*_^2^ mode is present.

We next discuss the frequency and linewidth dependence of the modes shown in Fig. 5 as a function of inverse thickness of the samples. Figure 5a and c (Fig. 5b and d) show the mode frequency and linewidth dependences, respectively, in the absence (presence) of the A_1*u*_^2^ mode in the Raman spectra. In Fig. 5a and b, the A_1*g*_^1^ stretching mode frequency exhibited the least dependence on *1/t* while the E_*g*_^2^ pinch mode frequency increased slightly with increasing *1/t*. Their linewidth dependences were relatively weaker than that exhibited by A_1*g*_^2^ and A_1*u*_^1^ modes. A_1*g*_^2^ mode is most sensitive to *1/t*: its frequency blueshifted by ~3 cm^−1^ in Fig. 5a while it redshifted in Fig. 5b and exhibited a significant sharpening from ~21 to 7 cm^−1^ with increasing *1/t* (decreasing thickness). A blueshift of the A_1*g*_^2^ peak frequency with decreasing layer thickness was also reported by Zhao *et al*.^35^ for CVD-grown pristine Bi_2_Te_3_ (inset in Fig. 5a) although it was accompanied by a broadening of the peak.

The discrepancy between our results and those from Zhao *et al*. can be explained by considering the effect of Se. The A_1*g*_^2^ phonon mode in Bi_2_Te_3_ exhibits strong electron phonon coupling (EPC), which becomes stronger with doping^36^. The strong EPC is also responsible for the formation of a Kohn anomaly at the Brillouin zone center and the observation of Dirac fermions in the topological surface states^37^. For a phonon with strong EPC, the anharmonic contribution to phonon decay is dwarfed by decay into electron hole pairs. However, in doped systems where the Fermi level is greater than the phonon energy, Pauli blocking reduces the number of electron states for the phonon to decay into, resulting in a longer phonon lifetime. This is manifested as a sharpening of the Raman peak. In addition, doping-induced change in the Fermi surface moves the Kohn anomaly away from the center of the Brillouin zone, where Raman active phonons are probed, and consequently causes a stiffening of the phonon mode. Indeed such observations have been made in doped graphene and metallic carbon nanotubes^38–40^, where the E_*g*_^2^ mode (G peak) blueshifts and sharpens upon both hole and electron doping. While the observations of strong EPC and Kohn anomaly in *n*-type Bi_2_Te_3_ have been made only at low temperatures (<20 K), it is possible that the C/ME process breaks apart the Bi_2_Te_2.7_Se_0.3_ layers into Se-doped quintuples where Se is substituted at the Te^2^ sites in the Bi_2_Te_3_ quintuple, maintaining the crystal symmetry. In that case one could expect an increase in surface states especially with decreasing layer thickness, consistent with our observation of blueshifted peak frequencies (Fig. 5a) and decreasing linewidths of the A_1*g*_^2^ mode (Fig. 5c) with its strong EPC. This result also corroborates the increase in electron densities and formation of charged boundaries surmised from the transport measurements shown in Fig. 3.

Conversely, in the spectra where the A_1*u*_^2^ mode was observed (Fig. 4c), the A_1*g*_^2^ peak exhibited a redshift (by ~4 cm^−1^) and broadening (by ~5 cm^−1^), opposite to the trends seen in the spectra in which the A_1*u*_^2^ mode was absent. As mentioned above, the appearance of the A_1*u*_^2^ mode is attributed to breaking of crystal symmetry from the C/ME process. In this case it is possible that the C/ME process caused individual quintuples to fragment into sub-quintuples and the appearance of the A_1*u*_^2^ mode is induced by the disorder caused by this fragmentation. Due to spot-to-spot variations we did not observe any dependence of the A_1*u*_^2^ mode intensity on *1/t*. The Raman modes did exhibit broadening (by ~2–4 cm^−1^) with increasing *1/t* in the spectra when the A_1*u*_^2^ mode was present in the spectra (Fig. 5d). A decrease in the sample thickness typically leads to broadening of peaks, and such a broadening was indeed observed in the A_1*g*_ peaks with decreasing Bi_2_Te_3_ layer thickness by Zhao *et al*.^35^. The observation of disorder-induced IR active modes supports our thermal measurements on the exf*nh*-SPS samples, where *κ* reduced compared to the bulk value with increasing exfoliation time.

**Figure 6:**
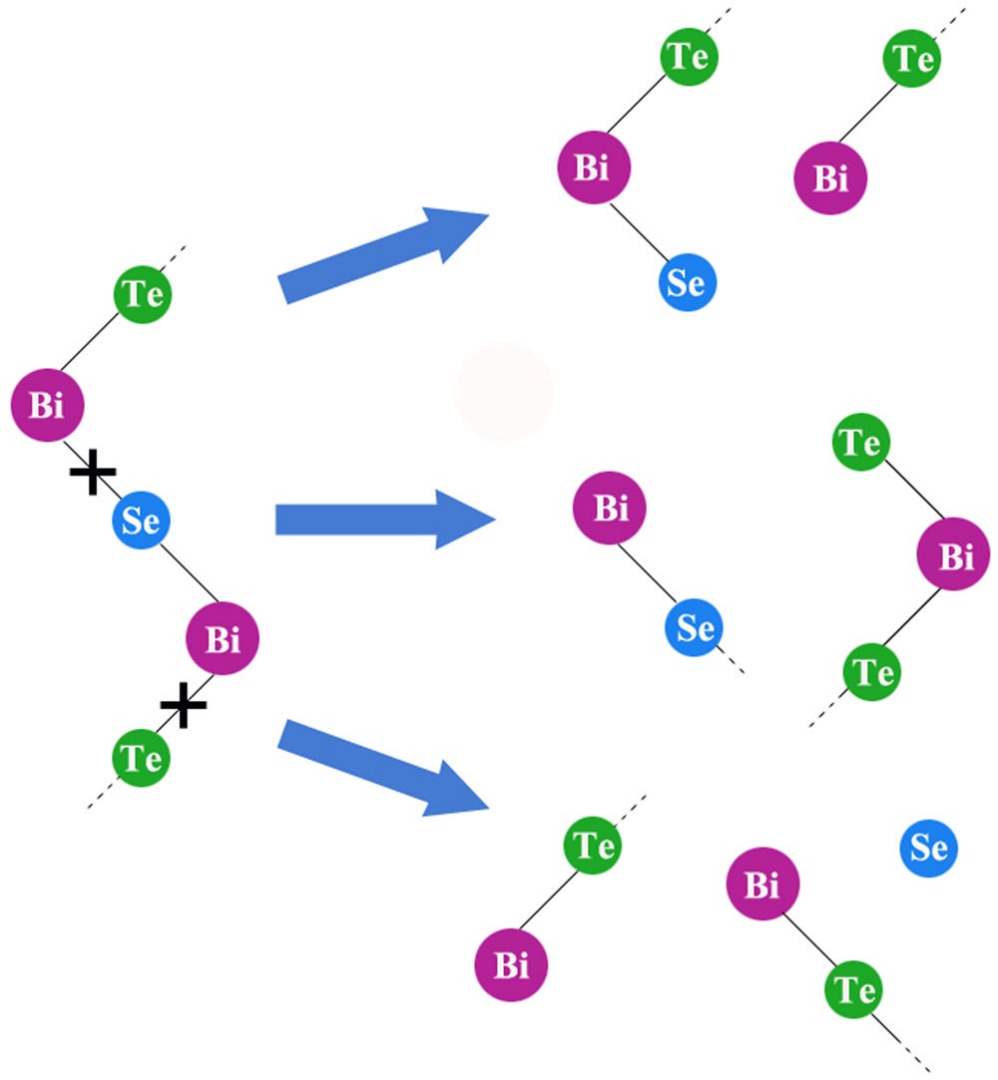
Schematic depiction of sub-quintuples formed as a result of the C/ME process.

Based on the Raman and transport measurements, a new picture of the structural changes that occur in the C/ME processed Bi_2_Te_2.7_Se_0.3_ crystal emerges: i) Se dopants preferably substitute for Te^2^ sites followed by Te^1^ sites, ii) during the chemical exfoliation process, the bond cleavages at various locations as depicted schematically in Fig. 6 (viz., Bi-Se or Bi-Te^1^ bond) to form different sub-quintuples such as Te^1^-Bi-Se^2^, Te^1^-Bi-Te^2^, Bi-Te^1^ and Bi-Se^1^, and iii) the co-existence of quintuples and sub-quintuples in the exfoliated samples (as evidence directly by AFM and indirectly by micro-Raman studies described in Figs 2 and 4), promotes the formation of charged grain boundaries during SPS treatment. The charged grain boundaries leading to the transport properties discussed in Fig. 3.

## Conclusion

In this study we presented systematic TE properties of few layered *n*-type Bi_2_Te_2.7_Se_0.3_ produced by C/ME-SPS process. The TE measurements showed an increase (decrease) in carrier concentration (resistivity and thermal conductivity), accompanied by a mitigation of bipolar effect in *α* in the exf*nh*-SPS samples. Systematic AFM and micro-Raman studies of C/ME processed samples provided evidence for the co-existence of quintuples and sub-quintuples, which upon SPS process promote the formation of charged grain boundaries. Specifically, micro-Raman analysis revealed two types of spectra, which exhibited different frequency and linewidth trends as a function of layer thickness. The first set of spectra provided evidence for scattering from Se-doped quintuples, where the increased EPC leads to stiffening and sharpening of the A_1*g*_^2^ phonon. In the second set of spectra the disorder-induced IR-active mode A_1*u*_^2^ was evident, which we attribute to the formation of sub-quintuples caused by the C/ME process.

## Methods

### Sample preparation

Pieces of *n*-type Bi_2_Te_2.7_Se_0.3_ ingot (Marlow Industries, USA) were dispersed in N-methyl-2-pyrrolidinone (NMP) with a ratio of 10 g/L and sonicated using 1/8-inch tip sonicator (Branson 250) at 20 W for 0, 3, 5 and 8 hrs to obtain Bi_2_Te_2.7_Se_0.3_ flakes. Subsequently, the supernatant solution was centrifuged at 4000 rpm for 2 hrs and the resulting powder was washed several times using deionized water to remove residual NMP and then oven dried at ~100 °C. Next, the exf*nh* flakes were compacted using spark plasma sintering (SPS, Dr. Sinter LabH-515S system) by loading ~2–3 g of exf*nh* into graphite dies and sintered at 500 °C for 5 minutes at an applied pressure of 30 MPa under a dynamic vacuum. The resulting SPS pellets were 12.5 mm in diameter and 2–3 mm in thickness, and a density of ~98–99% of the theoretical density. We refer to the SPS densified exf*nh* and commercial *n*-type Bi_2_Te_3_ ingot samples as exf*nh*-SPS and bulk, respectively.

### Characterization

The thicknesses of the exf*nh* flakes were measured using non-contact mode atomic force microscopy (AFM, Model: AIST-NT Smart SPM, Micromasch cantilevers HQ: NSC14/Al BS-50). The average thickness was statistically calculated from the AFM height measurements conducted on 200–400 flakes for each exf*nh* sample. In addition, two-pass Kelvin probe force microscopy (KPFM, Micromasch conductive AFM probes HQ:NSC14/Cr-Au, scan rate: 1.0 Hz) was used for imaging and measuring the contact potential difference between the AFM tip and the sample at the charged grain boundaries. For each scan line, during the two-pass KPFM measurement, the height profile was recorded as AFM topographic image and followed by lifting the probe by 30 nm above the surface to measure the potential offset. The AIST-NT image analysis and processing software (Version 3.2.14) was used for AFM topographic and KPFM image analysis. The microstructural and chemical analyses were performed using the conventional transmission electron microscopy (TEM, Hitachi H7500) and high resolution X-ray diffraction (HR-XRD, RIGAKU Ultima IV diffractometer, Cu K*α* radiation, *λ* = 1.5406 Å). Micro-Raman spectroscopy of exf*nh* samples was performed using a 633 nm excitation in a Renishaw Raman microscope equipped with a 100x objective lens (600 nm spot size). A reduced laser power (~100 *μ*W) was used to prevent the inadvertent overheating of the exf*nh* flakes during the collection of their Raman spectra. Similar micro-Raman measurements were performed on the exf*nh*-SPS samples but the shiny surfaces of these samples did not yield a good Raman signal.

All transport measurements were performed in the perpendicular direction to the SPS pressing direction. The temperature dependent (15 to 300 K) resistivity and thermopower were measured quasi-simultaneously using a 4-probe measurement technique which is described in detail elsewhere^41^. The commercial ZEM (ULVAC-RIKO, ZEM-2) was used to measure resistivity and thermopower from 300–500 K under partial He-atmosphere. The thermal conductivity (*κ*_*T*_) was measured from 20–320 K, using a standard steady-state technique on a custom designed measurement system^42^. The high temperature thermal conductivity was calculated using the relation *κ*_*T*_ *= C*_*p*_ *Dd*; where *d* is the packing density of the material, *D* the thermal diffusivity and *C*_*P*_ (≈*C*_*V*_, for solids) the specific heat capacity. The heat capacity was measured using a NETZSCH DSC 404C, thermal diffusivity was measured using a NETZSCH LFA 457 system and the packing density was measured by the Archimedes' principle. Since the laser flash measures the thermal diffusivity along the SPS pressure direction, whereas the low temperature transport properties are measured along the direction perpendicular to SPS direction (in the plane of pellet), several bars of our samples were cut and re-stuck together after rotating the bars by 90 degrees using JB Weld (a thermally conducting and electrically insulating glue), in order to measure all the properties along the same direction. Both the electronic and thermal transport measurements in the low and high temperature regimes were in good agreement over the entire temperature range of 15–500 K. The carrier concentration was determined from 10–300 K by Hall coefficient measurements using the commercial Quantum Design Physical Properties Measurement System (PPMS) under a magnetic field sweep of 5 kOe.

## Electronic supplementary material

Supplementary Information
