# Supplementary material for: A micro-Raman study of exfoliated few-layered n-type Bi2 Te2.7Se0.3
Source: Sci Rep. 2017 Nov 28;7:16535. doi: 10.1038/s41598-017-16479-y (PMC5705651; doi:10.1038/s41598-017-16479-y)
Supplement: Supplementary file 1 — Supplementary Information [file 41598_2017_16479_MOESM1_ESM.pdf]

Supplementary Information

A micro-Raman study of exfoliated few-layered *n*-type $Bi_2Te_{2.7}Se_{0.3}$

Fengjiao Liu[1], Longyu Hu[2], Mehmet Karakaya[1], Pooja Puneet[1], Rahul Rao[3,4*],

Ramakrishna Podila[1,5], Sriparna Bhattacharya[1*] and Apparao M. Rao[1,5]

1. Clemson Nanomaterials Institute, Department of Physics and Astronomy, Clemson University, Clemson, SC 29634 USA
2. Department of Chemistry, Clemson University, Clemson, SC 29634 USA
3. Materials and Manufacturing Directorate, Air Force Research Laboratory, WPAFB, OH 45433 USA
4. UES Inc., Dayton, OH 45432 USA
5. Laboratory of Nano-biophysics, Clemson University, Clemson, SC 29634 USA

Email: Rahul.rao.ctr.in@us.af.mil, bbhatta@clemson.edu

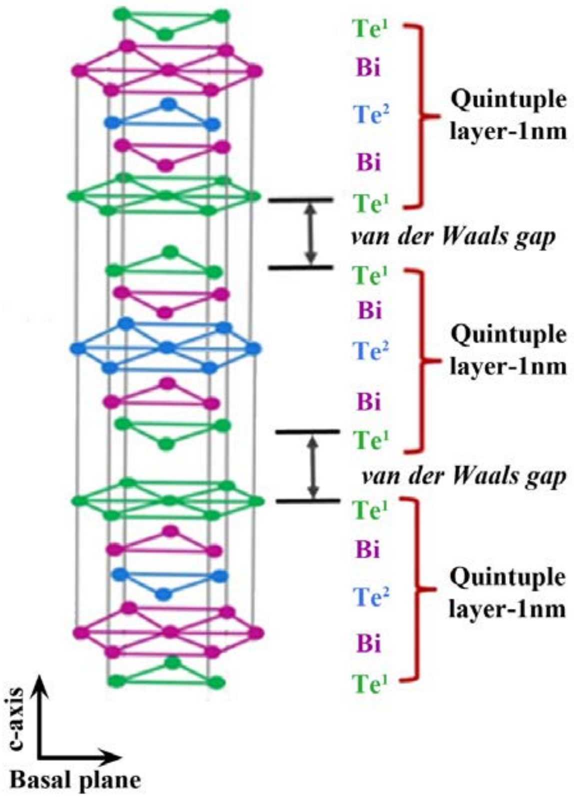

Supplementary Figure S1. Conventional hexagonal unit cell structure of pristine Bi_2Te_3

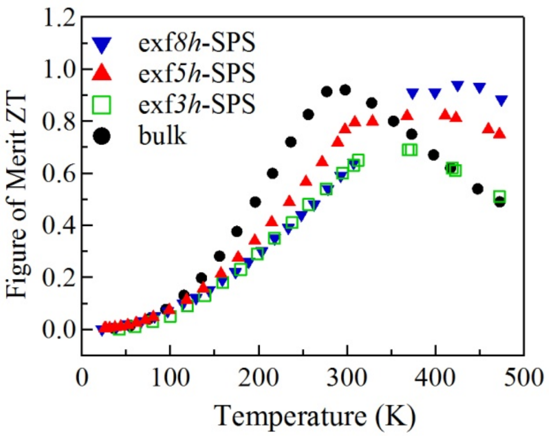

Supplementary Figure S2.The figure of merit *ZT* of *n*-type $Bi_2Te_{2.7}Se_{0.3}$ as a function of temperature

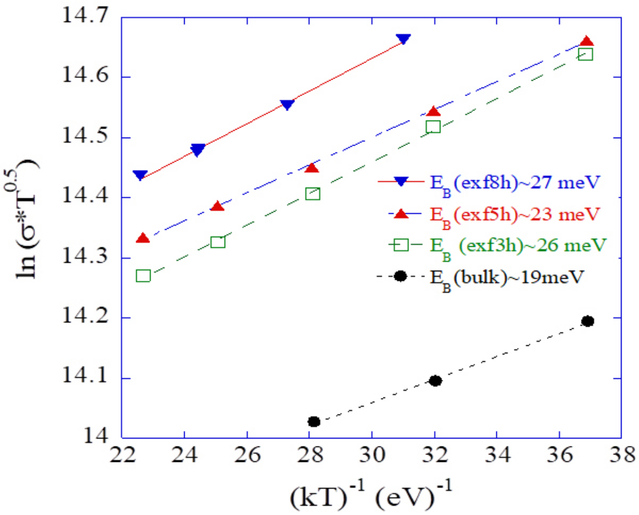

Supplementary Figure S3. Grain boundary potential barrier heights (E_B) of *n*-type $Bi_2Te_{2.7}Se_{0.3}$ samples determined from the linear plot of ln($\sigma * T^{1/2}$) *vs. 1/kT*.

Kelvin probe force microscopy (KPFM)

KPFM has been widely used for measuring the contact potential difference ($V_{CPD} = \frac{\varphi_{tip} - \varphi_{sample}}{-e}$) between its conductive AFM tip and the sample surface at the nanoscale level[1-4], where φ_{tip} and φ_{sample} are the work functions of the AFM tip and the sample respectively, and *e* is the electronic charge. The AFM phase image of a fractured exf*8h*-SPS sample surface (5 µm x 5 µm) is shown in Fig. S4a, where the different orientations of the grains give rise to grain boundaries (GBs). The corresponding KPFM CPD image of the same surface is shown in Fig. S4b, where the brighter contrast along the grain boundaries represents higher V_{CPD} values[3]. Representative height and V_{CPD} line profiles are shown in Figs. S4c-S4e and Figs. S4f-S4h, respectively. It is well known that in KPFM an abrupt change in height can also lead to a change in the measured V_{CPD}, as observed in Figs. S4c and S4f for the purple line profile. Similar changes in V_{CPD} are observed in Fig. S4g (S4h) due to changes in height, as indicated by the blue (orange) line profile in Fig. S4d (S4e). However, an increase in V_{CPD} ~ 30 mV across points 5 and 6 (10 and 11) which corresponds to the brightest section in the CPD image (Fig. S4b) is indicative of the presence of a charged GB[3].

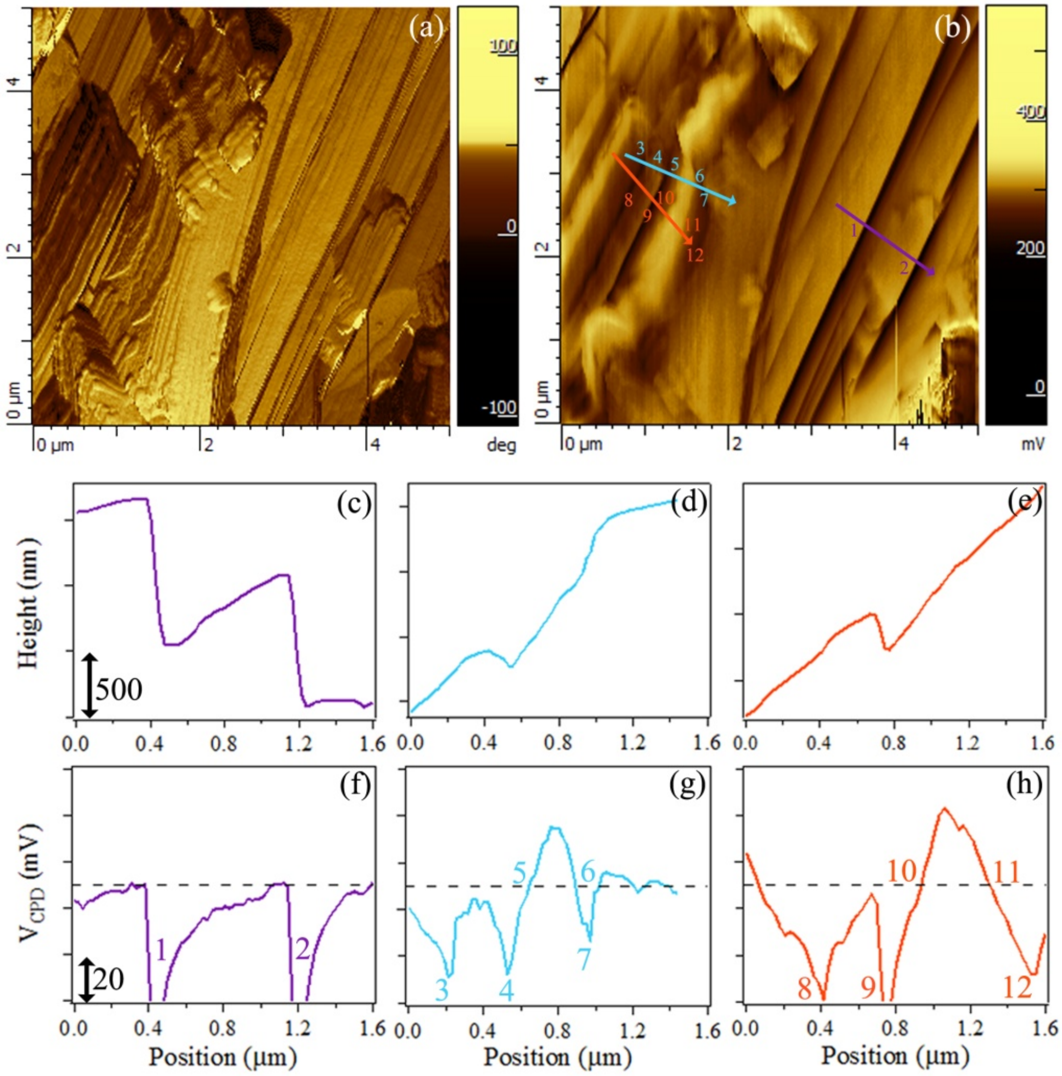

Supplementary Figure S4. KPFM of *n*-type exf*8h*-SPS $Bi_2Te_{2.7}Se_{0.3}$ fracture surface. (a) Phase image; (b) V_{CPD} image; (c-e) are the height changes along the purple, blue and orange line profiles in (a) and (f-h) are the corresponding V_{CPD}

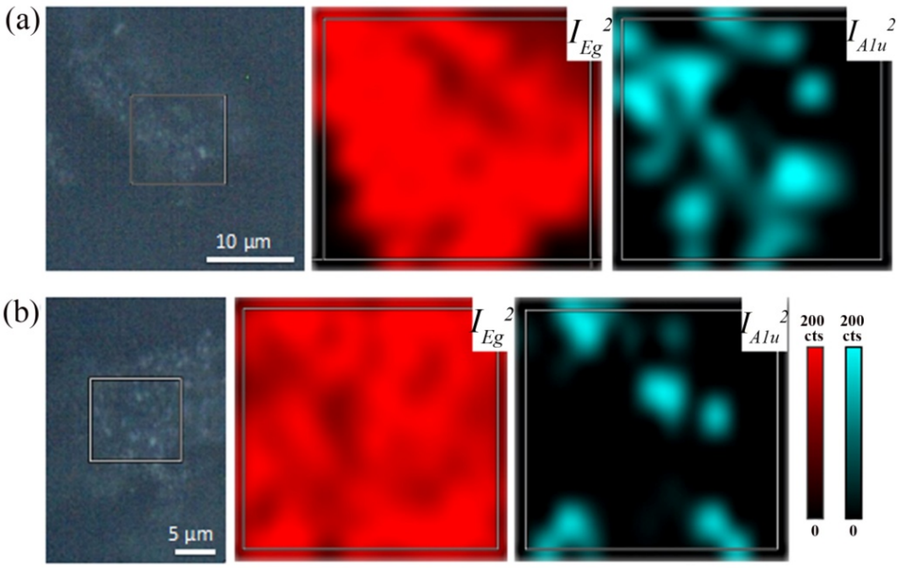

Supplementary Figure S5. (a) and (b) 2D Raman intensity maps collected from the square regions outlined in the optical microscope images. The maps were collected from aggregates of the 3 hour exfoliated $Bi_2Te_{2.7}Se_{0.3}$ flakes, deposited on to a glass slide. The E_g^2 peak is observed everywhere as evident from the red color intensity maps, while the A_{1u}^2 peak is observed at random locations (cyan map)

Supplementary Table S1 Calculated energy band gap (E_g) of *n*-type $Bi_2Te_{2.7}Se_{0.3}$.

Sample	α_{max} (μV/K)	T_{max}(K)	E_g(eV)
Bulk	222.58	362.0	0.161
exf *3h*-SPS	181.10	463.0	0.167
exf *5h*-SPS	181.10	463.1	0.168
exf *8h*-SPS	170.41	474.6	0.162

Supplementary Information References:

1. Stanford, M. G. *et al.* Focused helium-ion beam irradiation effects on transport properties of few-layer WSe_2: enabling nanoscale direct write homo-junctions. *Nature Scientific Reports* **6,** 27276, 1-10 (2016).
2. Hao, G. L. *et al.* Growth and surface potential characterization of Bi_2Te_3 nanoplates. *AIP Advances* **2,** 012114, 1-8 (2012).
3. Yan, Y. F. *et al.* Electrically benign behavior of grain boundaries in polycrystalline $CuInSe_2$ films. *Physical Review Letters* **99,** 235504, 1-4 (2007).
4. Melitz, W., Shen, J., Kummel, A. C. & Lee, S. Kelvin probe force microscopy and its application. *Surface Science Reports* **66,** 1-27 (2011).
